# Evaluation of the benefits of neutral bicarbonate ionized water baths in an open-label, randomized, crossover trial

**DOI:** 10.1038/s41598-024-51851-9

**Published:** 2024-01-13

**Authors:** Ryoko Ushikoshi-Nakayama, Tomoe Yamazaki, Daisuke Omagari, Naoyuki Matsumoto, Hiroko Inoue, Chiyoko Nukuzuma, Seiji Nishino, Ichiro Saito

**Affiliations:** 1 Department of Pathology, Tsurumi University School of Dental Medicine, 2-1-3 Tsurumi, Tsurumi-ku, Yokohama, Kanagawa 230-8501 Japan; 2 Department of Pharmaceutical Sciences, Nihon Pharmaceutical University, 10281 Komuro, Ina-machi, Kitaadachi-gun, Saitama, 362-0806 Japan; 3 Bicarbonate Thermotherapy Institute Co., Ltd., Park West Bldg. F7, 6-12-1 Nishishinjuku, Shinjuku-ku, Tokyo, 160-0023 Japan; 4 Psychiatry and Behavioral Sciences, Sleep and Circadian Neurobiology Laboratory, Stanford University School of Medicine, 3155 Porter Drive, Room 2016, Palo Alto, CA 94304 USA; 5 Cranescience Co., Ltd., 3-9-8 Ginza, Chuo-ku, Tokyo, 104-8139 Japan

**Keywords:** Health care, Physiology, Circulation, Blood flow

## Abstract

We previously demonstrated that neutral bicarbonate ionized water (NBIW) bathing enhances blood flow by bicarbonate ions and described the underlying mechanism. However, additional clinical investigation was warranted to investigate the efficacy of NBIW bathing. Hence, we performed a randomized, open-label, crossover trial to examine the effects of NBIW bathing on mental stress, sleep, and immune function. Participants who regularly felt stressed were randomly assigned to NBIW or regular bathing for 4 weeks. Mental stress was assessed with the Brief Job Stress Questionnaire (BJSQ) and the Profile of Mood States Second Edition; sleep quality, with the Pittsburgh Sleep Quality Index Japanese version (PSQI-J) and actigraphy; and immune function, with laboratory tests. PSQI-J scores and actigraphy sleep latency and bed out latency improved significantly more with NBIW bathing than with regular bathing (*p* < 0.05). Furthermore, NBIW bathing reduced both stress-induced fluctuations in CD4^+^ and CD8^+^ T cell counts and fluctuations in the naive to memory T cell ratio and neutrophil phagocytosis, indicating improved immune function. These findings suggest that daily NBIW bathing could improve mental stress, sleep quality, and immune function and bring about positive health effects in those who experience stress in their daily lives.

## Introduction

Hot water balneotherapy in water rich in minerals and bicarbonate has traditionally been used around the world^1^, and various studies have been performed to investigate the effectiveness of balneotherapy in trauma^2^, skin diseases^3^, and musculoskeletal disorders^4–7^. In recent years, studies have reported positive effects of balneotherapy on sleep and metal stress^4,8–10^.

Mental stress was believed to affect biological homeostasis, such as emotional regulation and hormone secretion, and recently, it has become clear that it also causes sleep disorders, depression, and cardiovascular disease and increases susceptibility to infectious diseases and cancer^11–13^. In Japan, the morbidity rate due to mental stress has been continuously increasing since the period of high economic growth in the late 1950s, with over 60% of employees reported to be experiencing mental anxiety and stress^14^.

The suicide rate in Japan is by far the highest among the Group of Seven industrialized nations, and the leading causes have been reported to include anxiety and depression related to mental stress in the workplace^15^. Despite a modest decrease in the number of suicides after the enactment of the Basic Act on Suicide Prevention by the Japanese Government in 2006, the annual number of suicides remains high at 20,000^16^. In 2015, the Japanese Ministry of Health, Labour and Welfare mandated that workplaces conduct occupational stress checks on their employees^17,18^. Nevertheless, the effectiveness of this measure is limited because of the small number of industrial physicians affiliated with workplaces and the lack of methods for successfully coping with mental stress.

The COVID-19 pandemic deeply affected the lives of people globally, not only because of the physical health risks of infection, but also because of the considerable mental stress related to the significant pandemic-related lifestyle changes, which in turn posed a threat to mental health and increased suicide rates^19^. Against this background, the establishment of specific coping strategies to relieve stress is considered to be one of the most critical societal challenges^20^.

Previously, we performed a study on a neutral bicarbonate ionized water (NBIW) bath tablet. In contrast to hot springs, in which the water composition varies depending on area and weather conditions, the quality of our bathing tablet is stable, and people can enjoy balneotherapy at home without having to visit a hot spring. Our previous study showed that bathing in NBIW tends to increase blood bicarbonate ion concentrations and increase blood flow by increasing expression and phosphorylation levels of endothelial nitric oxide synthase and levels of nitric oxide (NO) in femoral vascular tissue, effects that are associated with enhanced blood flow^21^. Moreover, in a preliminary randomized controlled trial conducted on the basis of those experiments, we found that the effects of higher body temperature upon waking and one hour after bathing tended to occur earlier in the intervention than in the control group. When a mood states profile was used to evaluate the effectiveness of bathing in NBIW on stress improvement, vigor/activity scores were improved^21^. The earlier study did not evaluate effects on immunity, but the positive effects of balneotherapy on immunity have received much attention in recent years, and many studies have evaluated them^6,7,22^. Therefore, we conducted a randomized, open-label, crossover study to investigate the effect of NBIW on sleep, mental stress, and immune function. In particular, to more accurately evaluate the efficacy of NBIW in improving mental stress, we assessed sleep quality, which correlates with stress, not only subjectively with a questionnaire-based analysis, but also objectively with an activity meter.

## Results

### Study population

**Figure 1:**
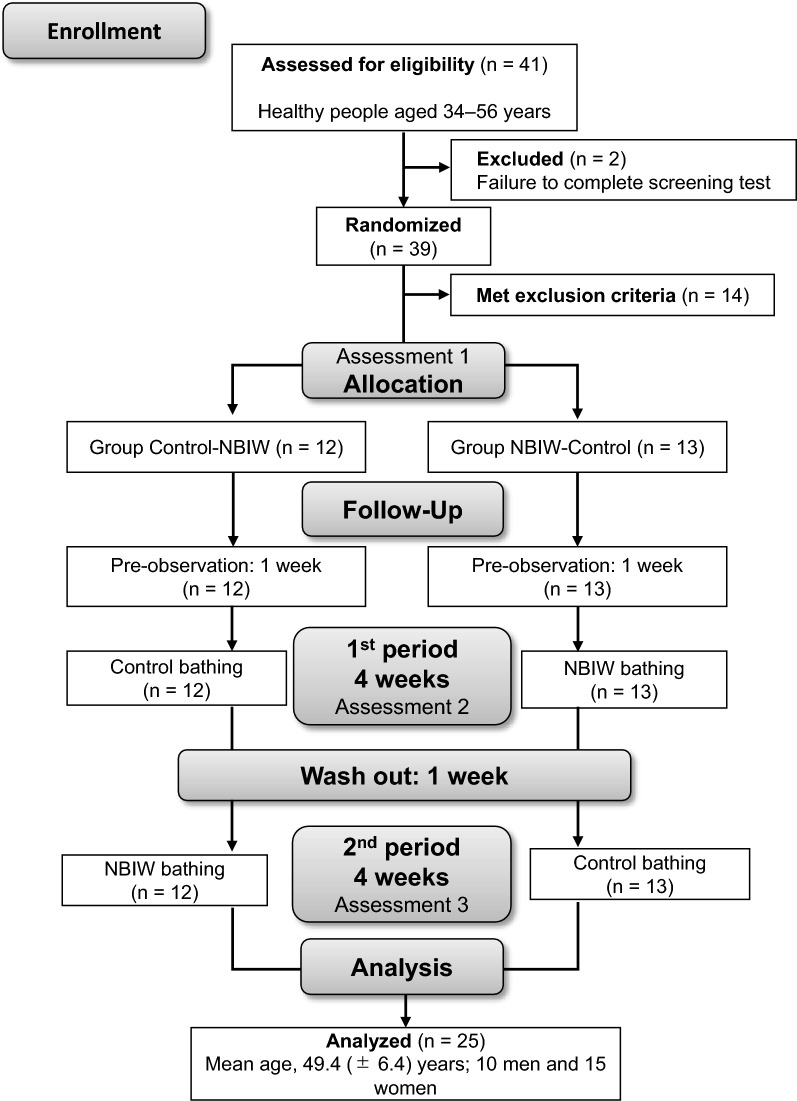
Flow diagram of the randomized crossover-controlled trial. NBIW, neutral bicarbonate ionized water.

A flow diagram of the study population is shown in Fig. 1. Of 41 potential participants who understood the content of the study and provided written informed consent, 39 completed the screening test, which comprised a simplified version of the Brief Job Stress Questionnaire (BJSQ) consisting of 23 questions^23^, the Pittsburgh Sleep Quality Index Japanese version (PSQI-J)^24^, age, mean hours of sleep, height, weight, body mass index, blood pressure, and pulse rate. Of those 39 individuals, 25 met the inclusion criteria, did not meet the exclusion criteria, and exhibited no clinical abnormalities based on the results of the screening test, which included the results of the BJSQ and the PSQI-J. This study used a crossover design comprising NBIW bath tablet bathing (NBIW) and standard bathing (control). In one group, control bathing was conducted first (control-NBIW, n = 12), while in the other group, NBIW bathing was conducted first (NBIW-control, n = 13). All 25 individuals participated in and completed the study and were eligible for inclusion in the efficacy analysis. Outcomes of each parameter were measured before allocation and at completion of the intervention. After completion of the whole study period, a statistician independent from the study group performed the statistical analyses.

### Background characteristics

Participant background characteristics are shown in Table 1. Analyses of background characteristics conducted when participants were allocated to groups after the screening test revealed no significant differences in age, mean sleep duration, physical measurements, physiological tests, PSQI-J, or BJSQ between the 2 crossover groups, i.e., control-NBIW and NBIW-control. The percentage of NBIW tablets used (% ± SE) in relation to the number of days of use was not significantly different between the groups (control-NBIW, 99.70% ± 1.03%; NBIW-control, 100.00% ± 0.00%). Bathing time during the intervention was calculated from the diary data recorded by each participant, and results confirmed that there was no significant difference in bathing time between NBIW bathing and control bathing.

**Table 1:** Background characteristics of the participants.

Items	Mean (± SD)		Between-group comparison, *p* value
	Group control-NBIW (n = 13)	Group NBIW-control (n = 12)	
Age (years)	49.3 ± 5.1	49.5 ± 7.6	0.6418
Mean hours of sleep			
Weekdays	5.67 ± 0.62	5.83 ± 1.11	0.2905
Weekends	6.38 ± 0.98	6.62 ± 1.02	0.5525
Height (cm)	164.97 ± 10.28	162.65 ± 5.76	0.4888
Weight (kg)	57.78 ± 11.39	63.63 ± 13.51	0.2554
Body mass index (kg/m^2^)	21.02 ± 2.00	23.92 ± 4.12	0.0502
Systolic blood pressure (mm Hg)	112.6 ± 19.1	120.0 ± 8.7	0.2360
Diastolic blood pressure (mm Hg)	72.4 ± 14.3	74.8 ± 8.7	0.6106
Pulse rate (bpm)	69.6 ± 9.9	72.0 ± 8.6	0.5214
BJSQ score			
A: Job stressors	18.3 ± 2.5	18.8 ± 2.9	0.6344
B: Stress reaction	25.0 ± 6.7	26.2 ± 5.2	0.6346
C: Social support	16.8 ± 2.6	16.5 ± 3.2	0.8025
PSQI-J total score	9.3 ± 1.8	9.4 ± 1.4	0.8332
Bathing time during the intervention (min)	NBIW (n = 25)	Control (n = 25)	0.8269
	22.8 ± 3.8	22.7 ± 3.5	

Between-group comparisons of age, mean hours of sleep, body mass index, and bathing time during the intervention were tested by Wilcoxon rank sum test; height, weight, diastolic blood pressure, pulse rate, Brief Job Stress Questionnaire, and the Pittsburgh Sleep Quality Index Japanese version, by Student’s *t* test; and systolic blood pressure, by Welch’s *t* test.

BJSW, Brief Job Stress Questionnaire; NBIW, neutral bicarbonate ionized water; PSQI-J, Japanese version of the Pittsburgh Sleep Quality Index.

### Outcomes

#### Stress

**Figure 2:**
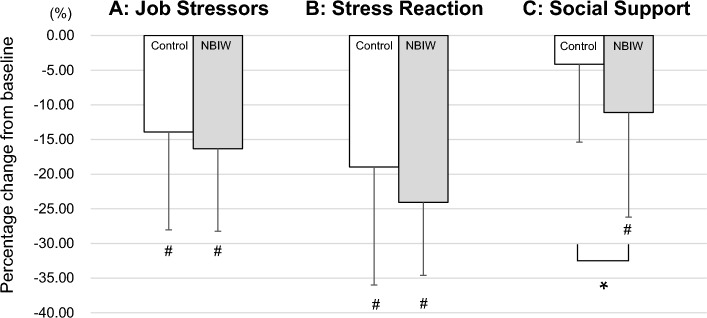
Assessment of stress with the Brief Job Stress Questionnaire. The Brief Job Stress Questionnaire (BJSQ), simplified version for primary outcome of mental stress, consists of 23 questions and was scored separately for the 3 stress categories. The graph shows the rate of change in scores from baseline (minus SD) on the vertical axis. A: control, − 13.92 (− 14.13); NBIW, − 16.32 (− 11.92). B: control, − 18.96 (− 17.03); NBIW, − 24.08 (− 10.53). C: control, − 4.14 (− 11.25); NBIW − 11.11 (− 15.08). N = 50 (control, n = 25; NBIW, n = 25). *p < 0.05 tested by paired *t* test. #: Parameters with significant changes from baseline (*p* < 0.05; tested by Wilcoxon rank sum test). NBIW, neutral bicarbonate ionized water.

**Figure 3:**
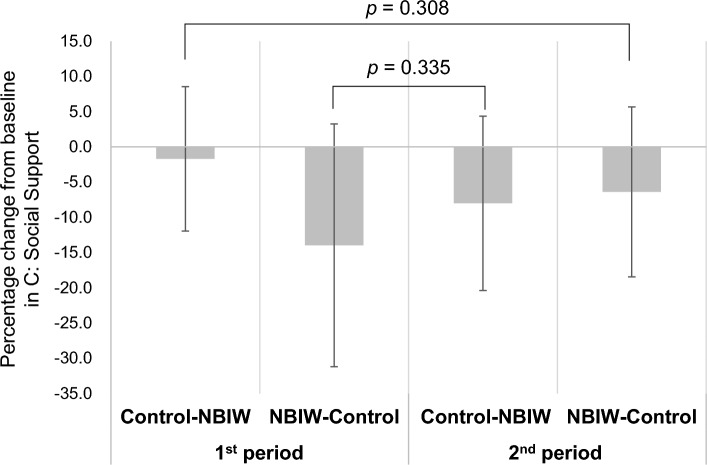
Test for carryover effect. For the percentage change from baseline in the Brief Job Stress Questionnaire-Social Support (BJSQ-C), the intervention periods were grouped into NBIW and control and compared with a *t* test. The first and second periods of the control intervention showed no significant difference (*p* = 0.308), and the same was found for the first and second periods of the NBIW intervention (*p* = 0.335). Thus, no carryover effect was observed.

The results of the BJSQ are shown in Fig. 2. Comparisons of percentage changes from baseline in 3 stressor categories (A, Job Stressors; B, Stress Reaction; and C, Social Support) revealed that the category C score decreased significantly more with NBIW bathing than with control bathing (*p* = 0.0193, d = − 0.52 [medium]). There was a significant difference in the BJSQ (category C score), one of the primary endpoints, between NBIW and control. The sample size was confirmed post hoc: The sample size estimated by power analysis with the BJSQ category C score was 25, and the power was 0.81. In addition, we confirmed that no significant carryover effect occurred (Fig. 3). Comparisons of the percentage change from baseline for each item in POMS2 are shown in Table 2. The item Confusion-Bewilderment decreased significantly more with NBIW bathing. A decrease in values or “Improvement in stress” were observed in both interventions in many items of the primary and secondary mental stress tests, but in both tests, the percentage changes were larger with the NBIW intervention than with the control intervention.

**Table 2:** Assessment of sleep quality by the profile of mood states 2nd edition.

Items		Percentage change from baseline					Within-group comparison	
		Control (n = 25), mean (± SD)	NBIW (n = 25), mean (± SD)	Between-group comparison			*p* value	
				*p* value	*r*	Effect size	Control	NBIW
AH (anger-hostility)	%	− 8.0 ± 14.7	− 9.6 ± 12.8	0.3341	− 0.14	Small	0.0083**	0.0014**
CB (confusion-bewilderment)	%	− 3.2 ± 11.9	− 10.0 ± 11.6	0.0478*	− 0.28	Small	0.1967	0.0005**
DD (depression-dejection)	%	− 6.1 ± 13.3	− 8.9 ± 12.1	0.2209	− 0.17	Small	0.0350*	0.0024**
FI (fatigue-inertia)	%	− 7.5 ± 13.4	− 13.0 ± 11.5	0.1064	− 0.23	Small	0.0090**	0.0001**
TA (tension-anxiety)	%	− 11.6 ± 13.5	− 14.2 ± 9.8	0.3651	− 0.13	Small	0.0007**	0.0000**
VA (vigor-activity)	%	15.1 ± 16.0	19.4 ± 21.4	0.3271	− 0.14	Small	0.0008**	0.0009**
F (friendliness)	%	1.4 ± 18.0	9.2 ± 17.8	0.0674	− 0.26	Small	0.5988**	0.0547
TMD (total mood disturbance)	%	− 10.4 ± 13.0	− 15.2 ± 9.9	0.0883	− 0.24	Small	0.0006**	0.0000**

Between-group and within-group comparisons were tested by Wilcoxon signed-rank test.

*p < 0.05, **p < 0.05.

Effect size was calculated as *r* family: small (0.1 ≤|*r*|< 0.3), medium (0.3 ≤|*r*|< 0.5), large (0.5 ≤|*r*|).

NBIW, neutral bicarbonate ionized water.

#### Sleep

**Figure 4:**
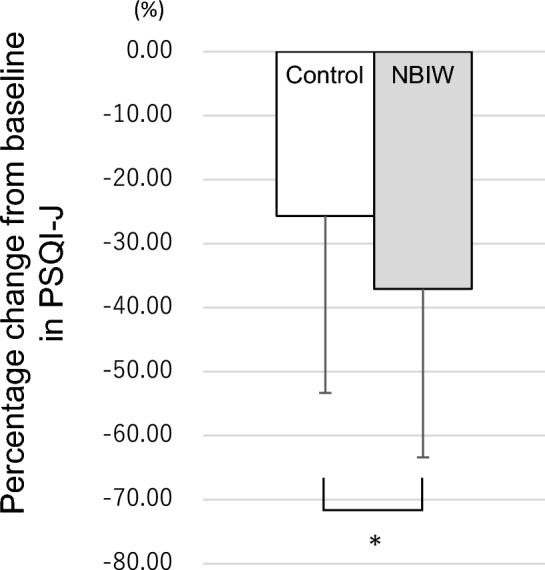
Sleep quality assessment with the Japanese version of the Pittsburgh Sleep Quality Index. The primary outcome *sleep quality* was assessed by the Japanese version of the Pittsburgh Sleep Quality Index. The graph shows the percentage change in scores from baseline (minus SD) on the vertical axis. Control, − 25.67% (SD, − 27.67%); neutral bicarbonate ionized water, − 37.08% (SD, − 26.33%). *p < 0.05 tested by paired *t* test. NBIW, neutral bicarbonate ionized water.

The baseline PSQI-J scores (mean ± SD) were 9.3 ± 1.8 in the control-NBIW group and 9.4 ± 1.4 in the NBIW-control group, i.e., in both groups, the scores indicated poor sleep quality (Table 1); however, with the NBIW intervention, scores improved, i.e., decreased (to 5.8 ± 2.4; Supplementary Information 1), and the percentage change from baseline was significantly lower in the NBIW intervention than in the control intervention (*p* = 0.0238, d = − 0.42 [small]) (Fig. 4).

Sleep was also analyzed by actigraphy, which provided data on bedtime, total sleep time, sleep latency, wake after sleep onset, sleep efficiency, time out of bed, and bed out latency. We observed that over 4 weeks, NBIW bathing generally improved sleep compared with control bathing. The results showed increased total sleep time and sleep efficiency and decreased waking after sleep onset, sleep latency, and bed out latency, and paired *t* tests showed that the decreases in sleep latency and bed out latency were significantly larger with the NBIW intervention than with the control intervention (Table 3). A power analysis of the items that showed significant differences between the interventions determined that a sample size of 27 participants would be necessary, assuming an effect size of 0.5, a probability of 0.05, and a power of 0.8. The post hoc calculations revealed larger effect sizes for sleep latency (1.43) and bed out latency (1.59).

**Table 3:** Evaluation of sleep quality by actigraphy.

Items	Control (n = 25), mean ± SD	NBIW (n = 25), mean (± SD)	Significant difference between groups
Total sleep time	318.44 ± 13.8	320.96 ± 13.47	
Sleep latency	14.24 ± 1.68	12.06 ± 1.28	*
Wake time after sleep onset	72.24 ± 7.88	68.12 ± 5.80	
Sleep efficiency	77.52 ± 1.84	78.85 ± 1.57	
Sleep period time	390.68 ± 16.57	389.08 ± 15.22	
Time in bed	412.74 ± 16.89	407.57 ± 15.13	
Bed out latency	7.82 ± 0.98	6.43 ± 0.71	*

Between-group comparisons were tested by a paired *t* test (one-tailed).

NBIW, neutral bicarbonate ionized water.

*p < 0.05.

### Immune functions

The results of the lymphocyte subset analyses are shown in Tables 4 and 5. The proportion of CD4^+^ cells, i.e., helper T cells, was significantly higher with the NBIW bathing intervention than with the control intervention (Table 4), whereas the concentration of CD8^+^ cells, i.e., cytotoxic T cells, was significantly lower with the NBIW intervention than with the control intervention (Table 5). In comparison, in the control intervention, the proportion of CD4^+^ cells was significantly lower and the proportion of CD8^+^ and number of CD8^+^ CD28^+^ cells, which represent the progenitor cells of CD8^+^ cells, were significantly higher (Tables 4 and 5). Although there was no marked change in CD4^+^ T cell counts in either intervention, CD8^+^ T cell counts showed a significant increase with the control intervention (Table 5). These results indicate that the post-intervention CD4^+^ to CD8^+^ T cell abundance ratio (CD4^+^:CD8^+^) was higher in the NBIW intervention (although the difference compared with the control intervention was not significant) and showed a significant decrease with the control intervention (Table 5). CD4^+^CD45RA^+^, i.e., naive T cells, significantly decreased with the NBIW intervention (Table 4), resulting in a significant decrease in the ratio of the naive to memory T cells ratio (N:M ratio) by the NBIW intervention (Table 5). At Week 4, the N:M ratio was not significantly different between NBIW and control. However, when compared with the baseline, it was increased by the control intervention and significantly decreased by the NBIW intervention (Table 5). The proportion of CD16^+^CD56^-^ cells, i.e., mature natural killer (NK) cells, decreased significantly in the NBIW intervention. At Week 4, CD16+CD56− (%) was not significantly different between NBIW and control. When compared with baseline, it decreased significantly in the NBIW intervention but showed no significant decrease in the control intervention (Table 4). The proportion of CD20^+^ cells, i.e., B cells, and the number of B cells increased significantly after both the NBIW and control interventions (Tables 4 and 5). The proportion of CD3^+^ cells, i.e., mature T cells, in total lymphocyte counts did not differ significantly between the interventions. Neutrophil phagocytosis activity and NK cell activity increased significantly with both interventions. However, mean neutrophil phagocytosis activity at Week 4 was higher with the NBIW intervention than with the control intervention. As a result, effects on neutrophil phagocytosis activity were greater with the NBIW intervention than with the control intervention (Table 6).

**Table 4:** Effect of the intervention on lymphocyte subset percentages.

			Control (n = 25), mean (± SD)	NBIW (n = 25), mean (± SD)	Between-group comparison (4 weeks)			Within-group comparison	
					*p*	*d* or *r*	Effect size	*p*	
								Control	NBIW
CD3^+^	%	Baseline	73.936 ± 6.476		0.3633	0.09	–	0.0044**	0.0834
		Week 4	72.460 ± 6.052	72.998 ± 6.561					
CD4 +	%	Baseline	51.861 ± 7.358		0.0395*	0.22	Small	0.0076**	0.6514
		Week 4	50.025 ± 6.382	51.563 ± 7.453					
CD4^+^CD45RA^+^	%	Baseline	43.172 ± 11.520		0.1166	− 0.14	–	0.9746	0.0062**
		Week 4	43.142 ± 12.323	41.448 ± 11.545					
CD8^+^	%	Baseline	12.326 ± 5.408		0.1815	− 0.17	–	0.0065**	0.8746
		Week 4	13.382 ± 5.743	12.416 ± 5.927					
CD8^+^CD28^+^	%	Baseline	73.261 ± 12.081		0.7982	− 0.04	–	0.2641	0.4926
		Week 4	72.256 ± 13.544	72.314 ± 12.797					
CD16^+^CD56^−^	%	Baseline	0.719 ± 0.431		0.1424	− 0.21	Small	0.1935	0.0017**
		Week 4	0.587 ± 0.405	0.500 ± 0.355					
CD16^−^CD56^+^	%	Baseline	4.456 ± 3.128		0.8823	− 0.02	–	0.7672	0.3817
		Week 4	4.570 ± 3.418	4.477 ± 2.966					
CD16^+^CD56^+^	%	Baseline	7.868 ± 3.198		0.3958	− 0.16	–	0.2406	0.7995
		Week 4	8.472 ± 2.885	8.008 ± 2.993					
CD20^+^	%	Baseline	15.623 ± 4.726		0.8625	− 0.01	–	0.0366*	0.0222*
		Week 4	16.698 ± 5.421	16.630 ± 5.203					

Distribution of CD3+, CD4+, CD4+CD45RA+, CD8+, CD16+CD56+, and CD20+ populations was parametric, and distribution of other populations was non-parametric.

Between-group and within-group comparisons were tested by a paired *t* test (for parametric data) and Wilcoxon signed-rank test (for non-parametric data).

*p < 0.05, **p < 0.01.

For parametric data, effect size was calculated as Cohen’s *d*: small (0.2 ≤|*d*|< 0.5), medium (0.5 ≤|*d*|< 0.8), large (0.8 ≤|*d*|); for non-parametric data, effect size was calculated as r family: small (0.1 ≤|*r*|< 0.3), medium (0.3 ≤|*r*|< 0.5), large (0.5 ≤|*r*|).

NBIW, neutral bicarbonate ionized water.

**Table 5:** Effect of the intervention on lymphocyte subset concentrations.

			Control (n = 25), mean (± SD)			NBIW (n = 25), mean (± SD)			Between-group comparison (week 4)			Within-group comparison	
									*p*	*d* or *r*	Effect size	*p*	
												Control	NBIW
Number of T cells	/µL	Baseline	1154.2 ± 281.7						0.3065	− 0.14	Small	0.4926	0.9893
		Week 4	1203.0 ± 328.3			1177.6 ± 352.9							
Number of CD4^+^ T cells	/µL	Baseline	805.4 ± 200.5						0.8191	− 0.03	–	0.7468	0.7064
		Week 4	819.0 ± 179.4			830.6 ± 266.8							
Number of CD8^+^ T cells	/µL	Baseline	198.0 ± 111.6						0.0395*	− 0.29	Small	0.0011**	0.7672
		Week 4	230.2 ± 131.3			204.7 ± 123.3							
CD4^+^:CD8^+^ ratio	–	Baseline	5.209 ± 2.667						0.1829	− 0.19	Small	0.0110*	0.9250
		Week 4	4.800 ± 2.985			5.935 ± 4.399							
Number of naive T cells	/µL	Baseline	349.4 ± 142.5						0.0614	− 0.26	Small	0.8401	0.1919
		Week 4	355.0 ± 139.2			343.5 ± 166.7							
Number of memory T cells	/µL	Baseline	456.0 ± 131.6						0.3529	0.15	–	0.5935	0.1065
		Week 4	464.0 ± 131.0			487.2 ± 171.4							
N:M ratio	–	Baseline	0.826 ± 0.354						0.1253	− 0.18	–	0.8578	0.0070**
		Week 4	0.833 ± 0.375			0.770 ± 0.334							
Number of CD8^+^CD28^+^ T cells	/µL	Baseline	145.8 ± 94.6						0.0561	− 0.27	Small	0.0071**	0.7982
		Week 4	168.6 ± 114.2			147.2 ± 99.7							
Number of B cells	/µL	Baseline	251.3 ± 114.8						0.1918	− 0.18	Small	0.0061**	0.0333*
		Week 4	283.6 ± 133.8			268.2 ± 107.8							
Number of natural killer cells	/µL	Baseline	125.5 ± 69.6						0.2108	− 0.18	Small	0.0979	0.3531
		Week 4	142.4 ± 69.2			130.4 ± 74.3							

The distribution of memory T cell population and naive to memory T cells ratio (N:M ratio) were parametric; distribution of other populations was non-parametric.

Between-group and within-group comparisons were tested by paired *t* test (for parametric data) and Wilcoxon signed-rank test (for non-parametric data).

*p < 0.05, **p < 0.01.

Effect size was calculated for parametric data as Cohen’s *d*: small (0.2 ≤|*d*|< 0.5), medium (0.5 ≤|*d*|< 0.8), large (0.8 ≤|*d*|), for non-parametric as r family: small (0.1 ≤|*r*|< 0.3), medium (0.3 ≤|*r*|< 0.5), large (0.5 ≤|*r*|).

NBIW, neutral bicarbonate ionized water; N:M ratio, ratio of naive to memory T cells.

**Table 6:** Neutrophil phagocytosis activity and natural killer cell activity.

			Control (n = 25), mean (± SD)	NBIW (n = 25), mean (± SD)	Between-group comparison (week 4)			Within-group comparison	
					*p*	*r*	Effect size	*p*	
								Control	NBIW
Neutrophil phagocytosis activity	%	Baseline	83.4 ± 2.4		0.1542	− 0.20	Small	0.0350*	0.0044**
		Week 4	84.1 ± 2.0	84.6 ± 2.2					
NK cell activity	%	Baseline	24.9 ± 13.8		0.7378	− 0.05	–	0.0000**	0.0001**
		Week 4	35.7 ± 11.8	34.9 ± 11.4					

Between-group comparisons were tested by Wilcoxon signed-rank test.

*p < 0.05, **p < 0.01.

Effect size was calculated as *r* family: small (0.1 ≤|*r*|< 0.3), medium (0.3 ≤|*r*|< 0.5), large (0.5 ≤|*r*|).

NBIW, neutral bicarbonate ionized water; NK, natural killer.

### Adverse events

A total of 10 adverse events were reported during the course of this study, 7 of which occurred during the control intervention. No significant differences in the occurrence of adverse events were observed between the 2 interventions. In addition, reports of abdominal pain, diarrhea (loose stools), weight loss, and elevated creatinine kinase during the use of NBIW were judged by the investigator to be not causally related to the study product. Fisher’s exact test did not find a significant difference in the incidence of adverse events between NBIW and control (P = 1.000, Effect size: φ = 0.0576). No severe or serious adverse events or adverse drug reactions were observed (Table 7).

**Table 7:** Incidence of adverse events and adverse drug reactions.

Item			Control	NBIW
Adverse events	Symptoms	Nausea	1	0
		Malaise	2	0
		Headache	1	0
		Skin reddening	1	0
		Nasal discharge	1	0
		Abdominal pain	1	0
		Abdominal pain, diarrhea (loose stool)	0	1
		Weight loss	0	1
		Increase in creatine kinase	0	1
	Severity	Mild	4	3
		Moderate	0	0
		Severe	0	0
	Incidence	Number of patients	4	3
		**Number of events**	**7**	**3**
Adverse drug reactions	Symptoms	–	–	–
	Severity	Mild	0	0
		Moderate	0	0
		Severe	0	0
	Incidence	Number of patients	0	0
		Number of events	0	0

Significant values are in bold.

## Discussion

Balneotherapy, i.e., the treatment of diseases by bathing, has been practiced since ancient times for therapeutic and medical purposes, and its therapeutic effects on cardiovascular and dermatological diseases have been documented^25–27^. Our previous double-blind, placebo-controlled study of NBIW and our in vitro and in vivo studies suggested that NBIW increases body temperature, promotes blood flow via nitric oxide production, and improves mental stress and sleep quality^21^. However, the placebo control contained magnesium sulfate and sodium sulfate, both of which are known to promote blood circulation; hence, warm bathing in the placebo control also showed mild effects in some analysis items, making it difficult to analyze the effectiveness of NBIW. Therefore, we conducted the present randomized, open-label, crossover trial to further investigate the effects of bathing in NBIW. The study included assessments of immune function because of the close correlation between stress, sleep, and immune function.

A number of previous studies reported on the efficacy of balneotherapy in improving mental stress and sleep disorders, and a large-scale randomized controlled trial (n = 362) conducted in 2016 in Chongqing, China, showed that balneotherapy is effective in reducing mental stress and sleep disorders and alleviating general health concerns^8^. Moreover, given that balneotherapy reduces levels of cortisol, a stress biomarker, some authors have suggested that it may be beneficial in controlling mental stress^28^. In the stress assessment in the present study, BJSQ categories A (Job Stressors) and B (Stress Reaction) were significantly improved after both the NBIW and control interventions. On the other hand, category C (Social Support) was significantly improved by the NBIW intervention but showed no change with the control intervention. For Category A and B, bathing itself is seen to be effective in reducing stress. Because there was a significant improvement also in category C with NBIW bathing compared with standard bathing without any additions, NBIW bathing is considered to be more effective in reducing stress. Moreover, the questions related to BJSQ category C evaluate whether a study participant receives sufficient support from those around them. We suggest that NBIW bathing improved study participants’ mental condition, which resulted in a change in their interpersonal cognition.

Balneotherapy over a period of 2 to 3 weeks may have beneficial effects on sleep quality^29^. It is thought to improve sleep by lowering systemic blood pressure and core body temperature by dilating peripheral blood vessels throughout the body, reducing pain by inhibiting inflammation and pain-related substances, and relaxing muscles^30^. The decrease in blood pressure occurs when the parasympathetic nervous system is dominant. Dominance of the parasympathetic nervous system is also considered to improve sleep quality. In fact, percutaneous stimulation of the parasympathetic nerve was reported to improve sleep quality in retired veterans suffering from post-traumatic stress disorder^31^. The PSQI-J, one of the primary sleep endpoints in this study, assesses subjective sleep quality, including insomnia; the total score ranges from 0 to 21, and a score of 6 or more indicates a potential sleep problem^24,32^. In the present study, the PSQI-J score confirmed that the NBIW intervention led to significantly greater improvements in subjective sleep quality than the control intervention. Furthermore, in the actigraphy sleep assessment, which was used as an objective assessment of sleep, the mean values of each item during the 3-week period showed a significant reduction in sleep latency and bed out latency and a trend towards improved sleep in many items with the NBIW intervention compared with the control intervention.

Mental stress and sleep quality are known to interact closely, and studies have shown that sleep quality is reduced in stressful environments^33,34^ and that stress can be reduced by improving sleep quality^35^. In this study, NBIW bathing was considered to improve the quality of sleep not only subjectively but also objectively. In our previous study, we demonstrated that the bicarbonate ions in NBIW affect endothelial cells, and through phosphorylation of endothelial nitric oxide synthase, promote synthesis of nitric oxide, which dilates blood vessels, leading to improved blood flow and temperature elevation. Moreover, 4-week NBIW bathing improved sleep quality according to PSQ-J score and reduced stress according to POMS2 scores^21^. Together with the result of this study, these findings indicate that NBIW may improve sleep quality and decrease stress by promoting blood circulation via production of nitric oxide and increase of body temperature.

A large number of studies have shown that mental stress increases the risk of a wide range of diseases and may be a risk factor for cancer and autoimmune diseases^36,37^. Mental stress is also particularly strongly associated with cardiovascular disease^38^. For example, several articles have reported that mental stress induces myocardial ischemia in patients with coronary artery disease^39,40^ and that depression is correlated with cardiovascular disease^41^. In addition, the Japan Collaborative Cohort Study for Evaluation of Cancer Risk (JACC study), which was sponsored by the Ministry of Education, Science, Sports and Culture of Japan, identified mental stress as being associated with increased coronary artery disease and increased stroke mortality in women^42^, and in the large-scale international case–control INTERHEART study, psychosocial factors were found to more than double the risk of myocardial infarction^43^. One of the primary mechanisms underlying the onset of cardiovascular disease due to mental stress may be inflammation-mediated vascular endothelial dysfunction resulting from the release of inflammatory cytokines as a result of suppression of the parasympathetic nervous system^44^. A previous study showed that the bicarbonate ions in NBIW act directly on vascular endothelial cells to induce nitric oxide production through phosphorylation of endothelial nitric oxide synthase^21^. As such, these findings suggest that continued NBIW bathing with warm water may also reduce the risk of cardiovascular disease by improving both mental stress and vascular endothelial function.

Immune function is also thought to be closely related to stress and sleep^45,46^. Several studies have shown that stress is a risk factor for cancer and autoimmune diseases, suggesting that stress affects immune tolerance and anticancer immunity^37,47^. Furthermore, sleep deprivation is known to alter the secretion of inflammatory markers such as interleukins, tumor necrosis factor-α, other cytokines, chemokines, and acute phase proteins^48,49^.

In this study, changes in several immune factors were observed with NBIW and control bathing. The CD4^+^:CD8^+^ T cell ratio did not change significantly with the NBIW bathing intervention, but it decreased with the control bathing intervention because of an increase in CD8^+^ T cells. CD8^+^ T cells are known to fluctuate in number and function in response to stress^50–52^. These suggest that the CD8^+^ T-cell count decreased because NBIW bathing resulted in a decrease in stress.

The proportion of naive T cells, as represented by CD4^+^CD45RA^+^, decreased slightly after NBIW bathing, while the number of memory T cells showed increase, resulting in a lower N:M ratio. Thus, given that tissue-resident and circulating memory T cells play an essential role in anti-tumor immunity, NBIW bathing may enhance anti-tumor immune function^53^.

The proportion of CD16^+^CD56^-^ cells, a marker of mature NK cells, decreased significantly in the NBIW bathing intervention. CD16^+^CD56^-^ mature NK cells are increased by post-traumatic stress^42,54^, suggesting that NBIW bathing may reduce CD16^+^CD56^−^ mature NK cells by alleviating stress.

The proportion of CD20^+^ B cell markers among total lymphocytes, the number of B cells, and the level of neutrophil phagocytosis and NK cell activity increased significantly after both interventions, suggesting that warm bathing itself improves immune function. However, the mean value of neutrophil phagocytosis activity after 4 weeks was higher with NBIW bathing than with control bathing, and the change was greater with NBIW than with control.

This study has some limitations. First, most of the participants were middle aged. Second, people with self-perceived daily stress were recruited. Third, stress is subjective. These factors may limit the generalizability of the results.

Taken together, the results of this study suggest that NBIW bathing has a positive influence on stress, sleep, and immune function. Stress, sleep, and immune function interact with each other^45,46^, so the effect of NBIW on immune function may be mediated through the improvements in stress and sleep. In addition, given that bicarbonate ions stimulate nitric oxide production in macrophage cell lines stimulated by lipopolysaccharide and interferon-gamma, which in turn promotes inflammatory responses^55,56^, it is conceivable that increased bicarbonate ions in the blood due to NBIW bathing may have a direct effect on immune system cells. In the future, further studies of such mechanisms are warranted.

## Materials and methods

### Participant eligibility and recruitment

This randomized clinical trial was conducted and reported in compliance with the Declaration of Helsinki and the CONSORT Statement, respectively. It was approved by the Chiyoda Paramedical Care Clinic Ethics Review Board (IRB No.: 15000088; Approval No.: 22031805), and the protocol was registered with the UMIN-CTR (UMIN000047429) (07/04/2022). All participants provided written informed consent to participate in the study. The study was conducted between April 13, 2022, and August 11, 2022, and no major changes were made to the protocol during the study. Data were collected at Chiyoda Paramedical Care Clinic and other institutions, and statistical analyses were performed by the contract research organization, CPCC, Inc.

Participants were recruited by means of widespread dissemination of information at the study sites. Participation was voluntary. The inclusion criteria were as follows: (a) men and women aged 30 to 60 years at the time of informed consent, (b) individuals who reported experiencing daily stress, (c) individuals who were dissatisfied with their sleep, (d) women who were postmenopausal or had a regular menstrual cycle of 28 to 32 days, (e) individuals who were able to take a bath once a day during the study period, and (f) individuals who were able to receive a full explanation of the study, understand its contents, and give their written consent. The exclusion criteria included 14 items (see Supplementary Information 2) in consideration of their impact on the efficacy evaluation and the safety of the participants. The major exclusion criteria included individuals taking medicines or supplements that could potentially have an impact on the study; individuals with irregular lifestyle rhythms, such as shift workers; individuals who may experience loss of consciousness due to seizures; individuals who have experienced coarse oily skin due to bath salts; and individuals who had donated a certain amount of blood before the start of the study. Study investigators and others enrolled study participants who gave written informed consent, satisfied the inclusion criteria, did not meet the exclusion criteria, exhibited no clinical abnormalities based on the results of the screening test, and were deemed eligible for participation in the study, taking into account the results of the BJSQ^23^ and PSQI-J^24^. The allocation manager, who was independent of the study and analysis sites, used stratified randomization to allocate eligible participants to 2 groups based on age, sex, and BJSQ and PSQI-J scores at the time of the screening test. The parameters used for allocation were analyzed by an unpaired *t* test or a Wilcoxon rank sum test, and it was confirmed that there was no significant difference between the two groups.

Subsequently, an allocation list was prepared with group names and participant identification numbers.

### Study design and intervention

This study was designed as a randomized, open-label, crossover trial with 2 intervention periods, each lasting 4 weeks, with a 1-week washout period between. The duration of the study product use was set to 4 weeks because another study showed an effect after 4 weeks of study product use, and the washout period was set to 1 week by referring to that study^21^. The study findings at the start of the pre-observation period, 1 week before the start of the intervention, were used as the baseline data.

The study bath salts were NBIW tablets (HOTTAB Inc.) that were pH adjusted to release the maximum amount of bicarbonate ions when dissolved in warm tap water. The composition of the tablets is shown in Table 8. The tablets were white and weighed 15 g each; once participants had opened the package, they were asked to store the tablets in a cool, dark place with low humidity.

**Table 8:** Ingredients of the neutral bicarbonate ionized water bath tablet.

Ingredients	%
Sodium bicarbonate	75
Sodium carbonate	5
Citric acid	15
Other	5

Participants were divided into 2 groups: after the first 1-week pre-observation period, one group bathed without NBIW tablets (control-NBIW group) and the other bathed with NBIW tablets (NBIW-control group). Then, after a 1-week washout period, each group participated in the other intervention. During the NBIW bathing period, participants were instructed to add 4 NBIW tablets to their bath (amount of water: 180 L) once a day and to enter the bath at least 15 min after adding the tablets. During the control period (including the pre-observation and washout periods), no NBIW tablets or other bath products were to be added to the once-daily baths. The bath water temperature was to be kept at 37 °C to 41 °C, and the bath was to be a full-body bath (i.e., shoulder depth) lasting a minimum of 20 min. Participants were permitted to drink room temperature water while bathing but were not permitted to drink cold water or read. The bath water was to be changed daily.

### Outcome measures

#### Primary endpoints

Primary endpoints were mental stress assessed with the BJSQ (simplified version consisting of 23 questions^23^, subjective sleep quality assessed with the PSQ-J^24,32^, and sleep measurements assessed with an activity meter (actigraphy)^57,58^. To obtain objective sleep variables, the small, lightweight waist-worn actigraphy device MTN-221 (Acos, Co., Ltd.) was used. Participants wore this device on their waist all times except when bathing. Sleep parameters were recorded for 3 weeks, from 5 days after the start of the intervention through to 2 days before its completion. Sleep and wakefulness were analyzed by SleepSign^®^-Act Ver. 2.0 (Kissei Comtec Co., Ltd, Matsumoto, Japan), which relies on an algorithm that uses the activity and posture data recorded by the actigraphy device in a series of linked calculations^59^. This algorithm was used to evaluate bedtime, total sleep time, sleep latency, wake after sleep onset, sleep efficiency, time out of bed, and bed out latency.

#### Secondary endpoints

POMS2^60,61^ was evaluated as the secondary endpoint of mental stress. Among immunological tests, lymphocyte subset, neutrophil phagocytosis, and NK cell activity were analyzed by flow cytometry (BD, FACS Caliber). Interleukin (IL)-6, IL-8, and IL-12 in blood were measured by enzyme-linked immunosorbent assay (LBIS Human IL-6 ELISA kit, LBIS Human IL-8 ELISA kit; FUJIFILM Wako Pure Chemical Corporation: Human IL-12 ELISA kit; abcam).

The BJSQ, PSQ-J, and POMS2 were assessed at the time of screening and at the end of each intervention period, and lymphocyte subset analyses were performed at the beginning of the pre-observation period and the end of each intervention period.

In addition, anthropometric measurements (height, weight, body mass index, abdominal circumference), blood pressure and pulse rate, general hematologic tests, eosinophil count, and white blood cell counts were performed. Furthermore, from the day after the start of the pre-observation period until the end of the second intervention period, participants completed a daily log on a dedicated website that included information on whether or not they had used the study product, whether or not they had taken baths, the duration of full-body bathing, the time they went to bed and woke up, their diet, and their intake of medicines and supplements.

### Statistical analysis

Microsoft Excel (Microsoft Corporation), IBM® SPSS26 (IBM Japan), BellCurve for Excel (Social Survey Research Information Co. Ltd.), and G*Power 3.1^62^ were used for tabulation, graphing, and analysis.

Data were analyzed with appropriate methods depending on normality, distribution, or correspondence. A paired *t* test was used for parametric data, and Wilcoxon signed-rank test was used for non-parametric data. In the case of parametric methods (paired *t* test), the effect size was calculated as Cohen’s d, and in the case of non-parametric methods (Wilcoxon signed-rank test), it was calculated as r.

### Supplementary Information

Supplementary Information.

## Data Availability

The data used in this study are available from the corresponding author upon reasonable request.
