# Supplementary material for: Evaluation of the benefits of neutral bicarbonate ionized water baths in an open-label, randomized, crossover trial
Source: Sci Rep. 2024 Jan 13;14:1261. doi: 10.1038/s41598-024-51851-9 (PMC10787754; doi:10.1038/s41598-024-51851-9)
Supplement: Supplementary file 1 — Supplementary Information. [file 41598_2024_51851_MOESM1_ESM.pptx]

## Slide 1
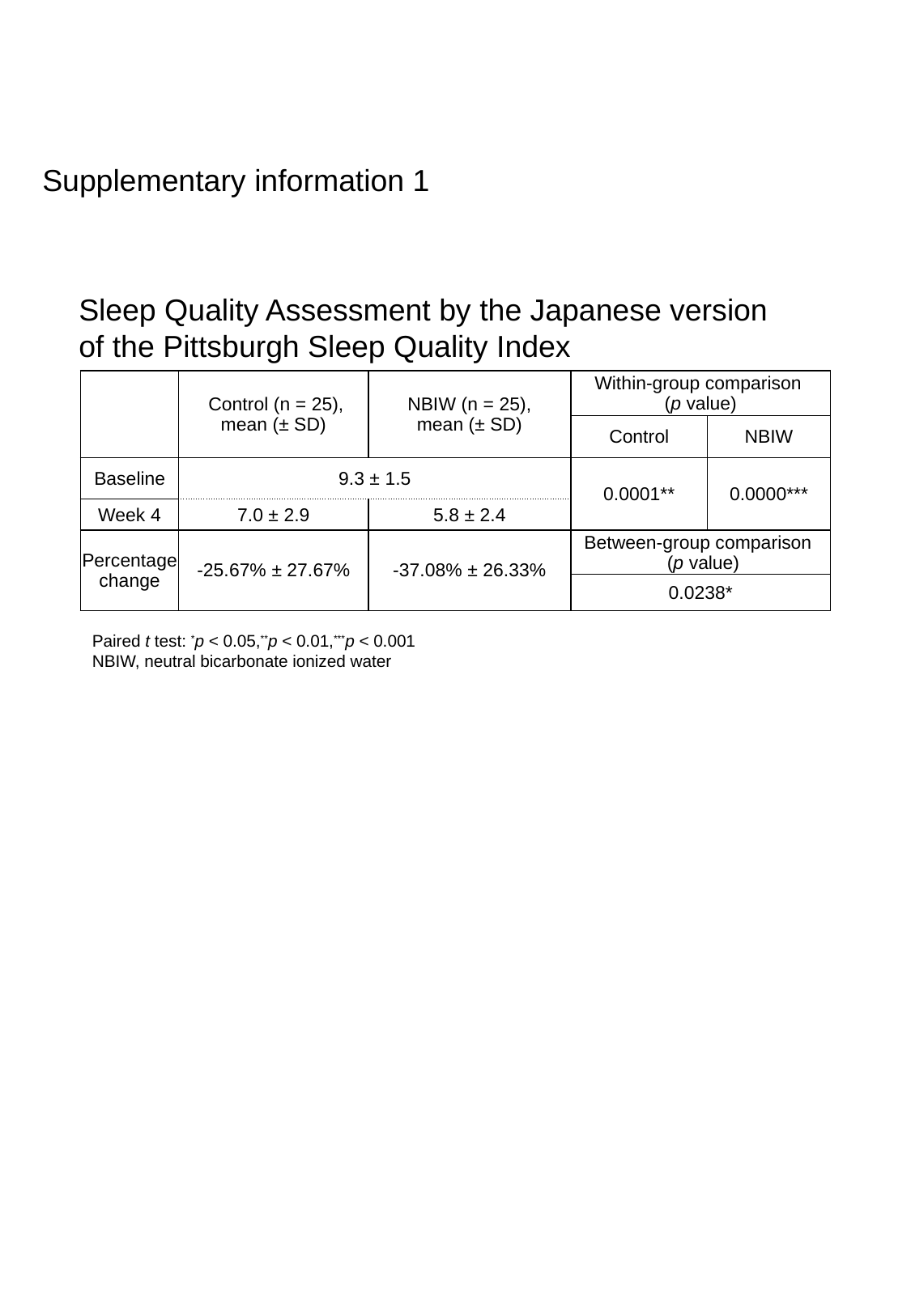

Supplementary information 1
Sleep Quality Assessment by the Japanese version of the Pittsburgh Sleep Quality Index
| | Control (n = 25), mean (± SD) | NBIW (n = 25), mean (± SD) | Within-group comparison (p value) | |
| --- | --- | --- | --- | --- |
| | | | Control | NBIW |
| Baseline | 9.3 ± 1.5 | | 0.0001\*\* | 0.0000\*\*\* |
| Week 4 | 7.0 ± 2.9 | 5.8 ± 2.4 | | |
| Percentage change | -25.67% ± 27.67% | -37.08% ± 26.33% | Between-group comparison (p value) | 0.6514 |
| | | | 0.0238\* | |
Paired t test: *p < 0.05,**p < 0.01,***p < 0.001
NBIW, neutral bicarbonate ionized water

## Slide 2
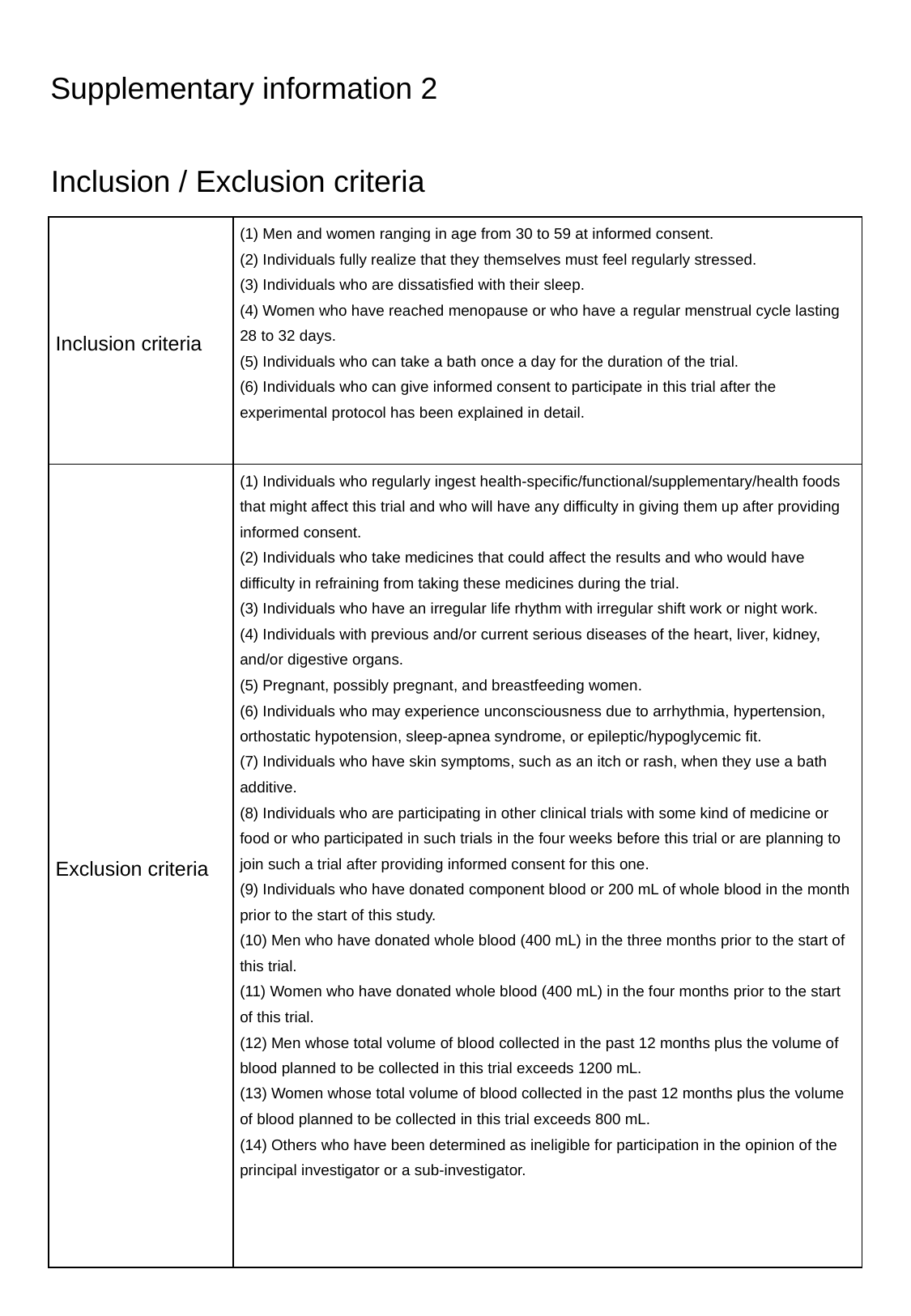

Supplementary information 2
Inclusion / Exclusion criteria
| Inclusion criteria | (1) Men and women ranging in age from 30 to 59 at informed consent. (2) Individuals fully realize that they themselves must feel regularly stressed. (3) Individuals who are dissatisfied with their sleep. (4) Women who have reached menopause or who have a regular menstrual cycle lasting 28 to 32 days. (5) Individuals who can take a bath once a day for the duration of the trial. (6) Individuals who can give informed consent to participate in this trial after the experimental protocol has been explained in detail. |
| --- | --- |
| Exclusion criteria | (1) Individuals who regularly ingest health-specific/functional/supplementary/health foods that might affect this trial and who will have any difficulty in giving them up after providing informed consent. (2) Individuals who take medicines that could affect the results and who would have difficulty in refraining from taking these medicines during the trial. (3) Individuals who have an irregular life rhythm with irregular shift work or night work. (4) Individuals with previous and/or current serious diseases of the heart, liver, kidney, and/or digestive organs. (5) Pregnant, possibly pregnant, and breastfeeding women. (6) Individuals who may experience unconsciousness due to arrhythmia, hypertension, orthostatic hypotension, sleep-apnea syndrome, or epileptic/hypoglycemic fit. (7) Individuals who have skin symptoms, such as an itch or rash, when they use a bath additive. (8) Individuals who are participating in other clinical trials with some kind of medicine or food or who participated in such trials in the four weeks before this trial or are planning to join such a trial after providing informed consent for this one. (9) Individuals who have donated component blood or 200 mL of whole blood in the month prior to the start of this study. (10) Men who have donated whole blood (400 mL) in the three months prior to the start of this trial. (11) Women who have donated whole blood (400 mL) in the four months prior to the start of this trial. (12) Men whose total volume of blood collected in the past 12 months plus the volume of blood planned to be collected in this trial exceeds 1200 mL. (13) Women whose total volume of blood collected in the past 12 months plus the volume of blood planned to be collected in this trial exceeds 800 mL. (14) Others who have been determined as ineligible for participation in the opinion of the principal investigator or a sub-investigator. |
